# Existing creatinine-based equations overestimate glomerular filtration rate in Indians

**DOI:** 10.1186/s12882-018-0813-9

**Published:** 2018-02-01

**Authors:** Vivek Kumar, Ashok Kumar Yadav, Yoshinari Yasuda, Masaru Horio, Vinod Kumar, Nancy Sahni, Krishan L. Gupta, Seiichi Matsuo, Harbir Singh Kohli, Vivekanand Jha

**Affiliations:** 1 Department of Nephrology, Postgraduate Institute of Medical Education and Research, Chandigarh, India; 2 Department of Nephrology, Nagoya University Graduate School of Medicine, Nagoya, Japan; 3 Department of CKD Initiatives, Nagoya University Graduate School of Medicine, Nagoya, Japan; 4 Division of Health Sciences, Graduate School of Medicine, Osaka University, Osaka, Japan; 5 Department of Dietitics, Postgraduate Institute of Medical Education and Research, Chandigarh, India; 6 George Institute for Global Health, 311-312, Elegance Tower, Plot No. 8, Jasola District Centre, New Delhi, 110025 India; 7 University of Oxford, London, UK

**Keywords:** Glomerular filtration rate, Chronic kidney disease, Creatinine, Cystatin C, Inulin clearance

## Abstract

**Background:**

Accurate estimation of glomerular filtration rate (GFR) is important for diagnosis and risk stratification in chronic kidney disease and for selection of living donors. Ethnic differences have required correction factors in the originally developed creatinine-based GFR estimation equations for populations around the world. Existing equations have not been validated in the vegetarian Indian population. We examined the performance of creatinine and cystatin-based GFR estimating equations in Indians.

**Methods:**

GFR was measured by urinary clearance of inulin. Serum creatinine was measured using IDMS-traceable Jaffe’s and enzymatic assays, and cystatin C by colloidal gold immunoassay. Dietary protein intake was calculated by measuring urinary nitrogen appearance. Bias, precision and accuracy were calculated for the eGFR equations.

**Results:**

A total of 130 participants (63 healthy kidney donors and 67 with CKD) were studied. About 50% were vegetarians, and the remainder ate meat 3.8 times every month. The average creatinine excretion were 14.7 mg/kg/day (95% CI: 13.5 to 15.9 mg/kg/day) and 12.4 mg/kg/day (95% CI: 11.2 to 13.6 mg/kg/day) in males and females, respectively. The average daily protein intake was 46.1 g/day (95% CI: 43.2 to 48.8 g/day). The mean mGFR in the study population was 51.66 ± 31.68 ml/min/1.73m^2^. All creatinine-based eGFR equations overestimated GFR (*p* < 0.01 for each creatinine based eGFR equation). However, eGFR by CKD-EPI_Cys_ was not significantly different from mGFR (*p* = 0.38). The CKD-EPI_Cys_ exhibited lowest bias [mean bias: −3.53 ± 14.70 ml/min/1.73m^2^ (95% CI: -0.608 to −0.98)] and highest accuracy (P_30_: 74.6%). The GFR in the healthy population was 79.44 ± 20.19 (range: 41.90–134.50) ml/min/1.73m^2^.

**Conclusion:**

Existing creatinine-based GFR estimating equations overestimate GFR in Indians. An appropriately powered study is needed to develop either a correction factor or a new equation for accurate assessment of kidney function in the Indian population.

**Electronic supplementary material:**

The online version of this article (10.1186/s12882-018-0813-9) contains supplementary material, which is available to authorized users.

## Background

Glomerular filtration rate (GFR) is the most important determinant for identifying and classifying chronic kidney disease (CKD) [1]. Since measuring GFR is impractical, equations that rely upon the serum concentration of filtration markers (i.e., molecules generated in the body at a fixed rate and eliminated almost exclusively by glomerular filtration) have been developed for estimating GFR (eGFR). Creatinine is the best known of these markers, and was the basis for the development of Modification of Diet in Renal Disease (MDRD) GFR estimating equation [2]. Recognizing that serum creatinine concentration was dependent upon muscle mass, demographic parameters that acted as surrogates for muscle mass, i.e. age, gender and ethnicity, are included in the equation. The MDRD equation was found to be biased and imprecise for values above 60 ml/min, and has been superseded by the Chronic Kidney Disease Epidemiology Collaboration (CKD-EPI) equation, developed in more diverse populations [3]. The 2012 Kidney Disease Improving Global Outcomes (KDIGO) guidelines recommend the use of this equation for diagnosis and follow up of CKD, as well as for screening of populations with unknown kidney function, unless equations developed in local populations are available [1].

Based on the concern that the ethnicity coefficient did not address nonwhite, non-African-American groups, investigations in Chinese and Japanese populations led to development of correction factors of 1.23 and 0.81, respectively [4, 5]. This meant that the same value of serum creatinine represented a 23% higher and 19% lower GFR compared to the white population, respectively. Correction factors or new equations have also been developed in Thai, Korean and Pakistani populations [6–8]. Since the largest differences are seen at higher GFR values, suggestions have been made to develop and validate ethnicity coefficients in populations where screening for CKD might occur.

Since muscle mass and dietary protein intake, in particular that of meat, are important determinants of serum creatinine concentration, it can be hypothesized that a correction factor, or a new eGFR equation may be necessary to accurately identify and classify individuals with CKD in the predominantly vegetarian Indian population, so that preventive and therapeutic strategies can be targeted appropriately. More recently, equations based on cystatin C (Cys), another filtration marker that is not dependent upon muscle mass and may be less susceptible to ethnic variations, have been developed [9]. Horio et al. showed that the CKD-EPI Cys (CKD-EPI_Cys_) eGFR equation developed in white population performed equally well in the Japanese [10].

In this pilot study, we evaluated the performance of the existing eGFR equations for assessment of GFR in Indians to determine the need for a larger study to develop an ethnicity coefficient for GFR estimation in Indians.

## Methods

### Study population

The study was done at the Postgraduate Institute of Medical Education and Research (PGIMER), Chandigarh, India. We studied participants of either sex over the age of 18 with normal kidney function (prospective living renal donors) as well as those with stable CKD. Organ transplant recipients, those on dialysis, edematous subjects, those with heart failure, current or past malignancy, history or current symptoms of voiding dysfunction, pregnancy, allergy to inulin, history of amputation in any limb, current use of glucocorticoids and current use of methyldopa were excluded. The study protocol adhered to the principles of the Declaration of Helsinki and was approved by the Institute Ethics Committee at PGIMER, Chandigarh. All participants provided written informed consent.

### Urinary clearance of inulin

GFR was measured by urinary clearance of inulin (C_in_). Enrolled participants reported for measurement of C_in_ after an overnight fast on the morning of completion of 24-h urine collection. Baseline anthropometric, demographic and clinical data were recorded. Participants were orally hydrated with 500 ml of water. Prior to start of continuous inulin infusion, the participants were instructed to void completely, and baseline blood and urine samples were collected. Inulin (Inulead®, Fuji Yakuhin Co Ltd., Saitama, Japan) was reconstituted in normal saline to 10 mg/ml as per manufacturer instructions, and infused at 300 ml/h. The infusion rate was decreased to 100 ml/h after 30 min. Participants voided completely after 30, 60 and 90 min of starting infusion. Blood samples were collected from the contralateral upper limb at 45 and 75 min. Participants were orally hydrated with 60 ml of water at 30 and 60 min. Infusion was stopped after the final complete voiding. The entire process was carried out under direct supervision. Serum and urine specimens were immediately stored at -80 °C until analysis. Inulin concentrations in serum and urine samples were measured by an enzymatic method using a kit (Diacolor Inulin, Toyobo Co, Osaka, Japan) as described before [5, 11, 12]. C_in_ was calculated as average of two timed collection periods and reported as measured GFR (mGFR). C_in_ measurement by two timed collection periods has been previously shown to be acceptable [13].

### Serum creatinine and cystatin C

Serum creatinine levels were measured by means of an isotope dilution mass spectrometry (IDMS) traceable assays both at PGI (modified Jaffe’s method using Cobas C111 auto-analyzer, Roche Diagnostics Limited) and in a central lab in Japan (enzymatic creatinine assay [5] using Hitachi creatinine auto-analyzer, model 7170, Hitachi). Serum Cys levels were measured in a central lab by colloidal gold immunoassay (Alfresa Pharma, http://www.alfresa-pharma.co.jp) which has been standardized for measurement of cystatin C traceable to ERM-DA471/IFCC [10].

### eGFR measurements

eGFR were calculated using serum creatinine values from enzymatic assay in following equations: serum creatinine based CKD-EPI_Cr_, Japanese coefficient-modified CKD-EPI [CKD-EPI (JAP)], MDRD and CKD-EPI Pakistan equation [CKD-EPI (PK)] and serum Cys based CKD-EPI_Cys_ (Additional file 1: Table S1) [3, 6, 9, 14, 15].

### Other measurements

Dietary habits were recorded as vegetarian or non-vegetarian (participants who consumed meat in any form). Dietary protein intake was estimated by 24-h urea nitrogen appearance calculated as sum of urine urea nitrogen (in grams/day) and non-urea nitrogen (calculated as weight in kg X 0.031 g kg/day) [16, 17]. Daily protein intake was calculated as urea nitrogen appearance multiplied by 6.25.

### Statistical analyses

Continuous data were presented as mean ± standard deviation with 95% confidence intervals (CI). Categorical data were presented as frequencies or proportions. Bias was expressed as mean difference between mGFR and eGFR (mGFR-eGFR), and expressed as mean ± standard deviation. 95% limits of agreement were calculated as mean bias ±1.96 x standard deviation. Precision was expressed as 95% confidence interval of the difference between mGFR and eGFR. Accuracy was expressed as root mean square error (RMSE) of difference between mGFR and eGFR, and percentage of participants with eGFR within ±30% of mGFR (P_30_). We performed sensitivity analyses using Jaffe creatinine values.

Continuous data were tested for normality and compared using independent sample t test if the data were normal. Mann Whitney U test was used if the data were not normally distributed. Correlation between serum creatinine levels done at PGIMER and in Japan was tested by Pearson’s correlation. Data were analyzed using the Statistical Package for the Social Sciences (SPSS) software for Macintosh, version 21.0 (IBM Corp., Armonk, NY, USA).

## Results

A total of 200 participants with stable CKD and 85 prospective renal donors were screened between January 2014 and December 2015. 150 participants were excluded (on dialysis: 63; organ transplant recipient: 15; edema: 22; heart failure: 11; voiding dysfunction: 8, use of glucocorticoids: 12; refused consent: 19). Finally, 135 participants were studied. Data collection were incomplete in 5 participants and hence, 130 participants were included for final analyses. These included 63 apparently healthy prospective living renal donors and 67 patients with CKD. The causes of CKD in these 67 individuals are shown in Table 1. The demographic, biochemical characteristics and GFR in study population are shown in Table 2. Majority were males and 50% were strict vegetarian. Among meat-eaters, the average frequency of meat intake was 3.8 times every month. The average weight and 24-h urine volume in study participants were 63.2 kg (95% CI: 61.0 to 65.3 kg) and 2321 ml (95% CI: 2135 to 2508 ml), respectively. The average daily urine creatinine and urea nitrogen excretion were 0.87 g (95% CI: 0.80 to 0.93 g) and 5.4 g (95% CI: 5.0 to 5.8 g), respectively. The creatinine excretion were 14.7 mg/kg/day (95% CI: 13.5 to 15.9 mg/kg/day) and 12.4 mg/kg/day (95% CI: 11.2 to 13.6 mg/kg/day) in males and females, respectively. The average daily protein intake based on 24-h urea nitrogen excretion was 46.1 g/day (95% CI: 43.2 to 48.8 g/day), respectively.

**Table 1:** Causes of CKD in study population with CKD (*n* = 67)

Cause of CKD	Number of participants
Unknown	27
Diabetic kidney disease	13
Chronic glomerulonephritis	9
Hypertensive nephrosclerosis	7
Chronic interstitial nephritis	5
Polycystic kidney disease	4
CAKUT	2

*CAKUT* Congenital abnormality of kidney and urinary tract, *CKD* Chronic kidney disease

**Table 2:** Characteristics and measurements in study population (*n* = 130)

Parameter	Total study participants (*n* = 130)
Age	44.62 ± 11.92
Sex (M/F)	72/58
Height (cm)	161.25 ± 10.82
Weight (kg)	63.17 ± 12.54
BMI (kg/m^2^)	24.62 ± 7.65
Hemoglobin (g/dL)	13.24 ± 1.97
Serum creatinine (mg/dL)	1.37 ± 0.88
Serum cystatin C (mg/dL)	1.75 ± 0.87
Blood urea nitrogen (mg/dL)	19.10 ± 11.67
Serum albumin (g/dL)	4.26 ± 0.40
Measured GFR by urinary inulin clearance (ml/min/1.73m^2^)	51.66 ± 31.68
Estimated GFR	
CKD-EPI_Cr_ equation (ml/min/1.73m^2^)	76.58 ± 41.05^a^
CKD-EPI (PK) equation (ml/min/1.73m^2^)	68.50 ± 38.40^a^
CKD-EPI (JAP) equation (ml/min/1.73m^2^)	62.26 ± 33.38^a^
MDRD equation (ml/min/1.73m^2^)	77.79 ± 43.36^a^
CKD-EPI_Cys_ equation (ml/min/1.73m^2^)	55.19 ± 33.58^b^

*BMI* body mass index, *CKD-EPI* Chronic Kidney Disease Epidemiology Collaboration, *Cys* cystatin C, *GFR* glomerular filtration rate, *F*: female, *JAP* Japan, *M* male, *MDRD* Modification of Diet in Renal Disease, *PK* Pakistan

^a^significantly different from measured GFR by urinary inulin clearance (*p* < 0.01 for each comparison)

^b^Not significantly different from measured GFR by urinary inulin clearance (*p* = 0.31)

Mann-Whitney U test used for comparison between measured GFR and estimated GFR

Expressed as mean ± standard deviation except sex

**Fig. 1:**
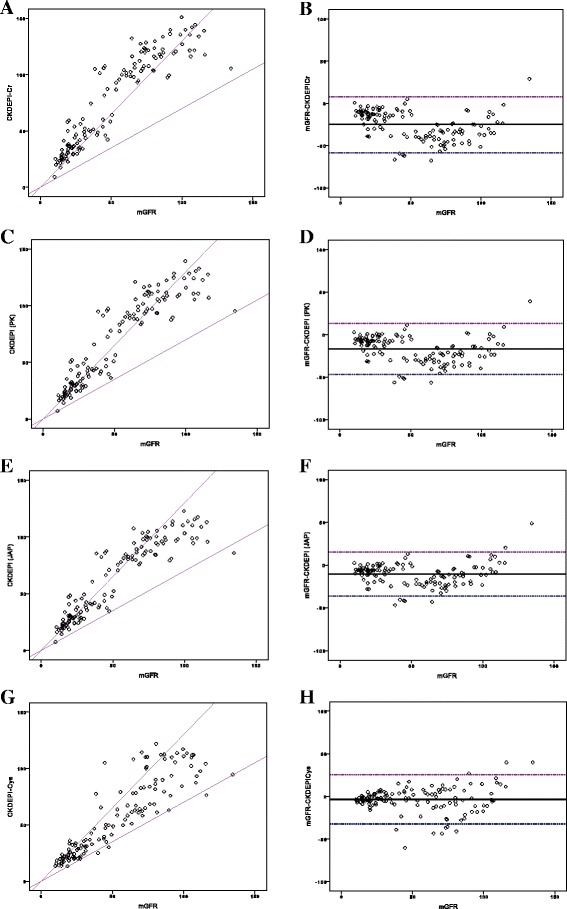
Accuracy of eGFR equations against mGFR. Comparison between mGFR and eGFR by CKD-EPI_Cr_ (**a**), CKD-EPI (PK) (**c**), CKD-EPI (JAP) (**e**) and CKD-EPI_Cys_ (**g**); the diagonal lines represent the values within 30% of mGFR Mean bias represented by solid line, and 95% limits of agreement (mean bias ±1.96 SD) represented by dashed lines for eGFR by CKD-EPI_Cr_ (**b**), CKD-EPI (PK) (**d**), CKD-EPI (JAP) (**f**) and CKD-EPI_Cys_ (**h**)

The mean mGFR in the study population was 51.66 ± 31.68 ml/min/1.73m^2^. A total of 54 participants had mGFR ≥60 ml/min/1.73m^2^ whereas 76 had mGFR <60 ml/min/1.73m^2^. Amongst apparently healthy participants (prospective renal donors), the mean mGFR was 79.44 ± 20.19 ml/min/1.73m^2^ (range: 41.90–134.50 ml/min/1.73m^2^). All five eGFR equations overestimated GFR (Table 3, Fig. 1), with CKD-EPI_Cr_ being the poorest. The CKD-EPI_Cys_ exhibited the lowest bias. The results did not change after stratification by gender (data not shown) or mGFR categories (Additional file 1: Tables S2 and S3) or age (Additional file 1: Tables S4 and S5). eGFR by any of the creatinine based estimating equations was significantly different from mGFR. Overall, the accuracy was best for CKD-EPI_Cys_ equation [Table 3, Fig. 1]. When stratified by mGFR groups, the proportion of participants with eGFR by CKD-EPI_Cys_ within ±30% of mGFR were 81.5% and 69.7% for those with mGFR ≥60 ml/min/1.73m^2^ and <60 ml/min/1.73m^2^, respectively.

**Table 3:** Performance of GFR estimating equations as compared to measured GFR by urinary inulin clearance

eGFR equation	Bias (mGFR-eGFR) (ml/min/1.73m^2^)	95% Limits of agreement (ml/min/1.73m^2^)	Precision (95% CI) (ml/min/1.73m^2^)	Accuracy	
				RMSE (ml/min/1.73m^2^)	P_30_ (%)
CKD-EPI_Cr_	−24.92 ± 17.17	−58.57 to 8.73	−27.90 to −21.95	30.22	22.3
CKD-EPI (PK)	−16.84 ± 15.39	−47.00 to 13.32	−19.52 to −14.17	47.72	39.2
CKD-EPI (JAP)	−10.62 ± 13.07	−36.22 to 15.02	−12.87 to −8.33	16.79	51.5
MDRD	−26.13 ± 22.74	−73.95 to 28.47	−30.09 to −22.17	34.63	25.4
CKD-EPI_Cys_	−3.53 ± 14.70	−32.34 to 25.28	−0.608 to −0.98	15.06	74.6

*CKD-EPI* Chronic Kidney Disease Epidemiology Collaboration, *CI* confidence interval, *Cys* cystatin C, *eGFR* estimated glomerular filtration rate, *F* female, *JAP* Japan, *M* male, *MDRD* Modification of Diet in Renal Disease, *mGFR* measured GFR, *PK* Pakistan, *P*_*30*_ percentage of participants with eGFR within ±30% of mGFR, *RMSE* root mean square error

Serum creatinine values measured by the modified Jaffe method at PGIMER, India strongly correlated with those done at the Central Laboratory in Japan (Pearson’s correlation coefficient *r* = 0.965, *p* < 0.001, Additional file 2: Figure S1). The mean difference between the two methods was −0.037 ± 0.243 mg/dL (*p* = 0.096) with 95% limit of agreement as 0.439 to −0.513, and no potential proportional bias (linear regression analysis: unstandardized B = −0.023, *p* = 0.336). Sensitivity analysis using eGFR calculated using the Jaffe creatinine showed similar performance (Additional file 1: Table S6).

## Discussion

This pilot study shows that the existing creatinine based eGFR equations significantly overestimate GFR in the predominantly vegetarian Indian population. Better performance of CKD-EPI_cys_ equation and lower creatinine excretion suggest that this overestimation is likely linked to the lower muscle mass. These findings support the need of an appropriately powered study to develop either a correction factor or a new equation for accurate assessment of kidney function in the Indian population.

In stable individuals, age, sex and race are important non-GFR determinants that affect creatinine generation and have been factored in creatinine based eGFR equations [18]. Most Indians are vegetarian because of cultural and religious beliefs. Even those who count themselves as non-vegetarians eat meat only infrequently. Also, the body habitus of individuals of Indian ethnicity is different from other Western and Asian populations. Indian individuals are comparatively short, have less muscle mass, and exhibit truncal obesity [19–22]. The measured daily creatinine excretion rates of 14.7 and 12.4 mg/kg/day in Indian males and females are lower than what has been reported for Japanese participants (20.2 and 16.7 mg/kg/day respectively) [5]. These important physiologic non-GFR determinants of serum creatinine have not been factored in the available serum creatinine based eGFR equations [18], and could account for poor performance of these equations in our study population, as shown by low P_30_ values.

This hypothesis was also supported by the better performance of cystatin-based equation. Notably, the eGFR by CKD-EPI_Cys_ and mGFR did not differ significantly in overall study population and GFR subgroups. Cys is produced by all nucleated cells and is less affected by age and sex [23–25]. Its generation is not dependent on muscle mass and dietary protein intake. In a study of 170 healthy individuals, serum creatinine and 24-h urinary creatinine excretion significantly correlated with lean body mass whereas serum Cys levels did not, even after adjustment for dietary protein/meat intake and level of physical activity [26]. This observation is important, as use of equations that use both serum creatinine and cystatin has been recommended to offset errors by equations that rely on individual parameters [27]. Similar finding has been reported amongst the Japanese, where the CKD-EPI_Cys_ accurately predicted mGFR, but addition of creatinine to the formula worsened the bias [10].

Another important finding was the relatively low mGFR in apparently healthy participants who were being worked up as kidney donors. This finding is consistent with earlier reports from India. In a selected population of 122 apparently healthy prospective renal donors, GFR measured by plasma clearance of technetium 99 m diethyltriaminepentaacetic acid was 83.42 ± 13.4 (range 61–130) ml/min/1.73m^2^ [28]. In another study of 101 healthy Indian adults, mGFR was 82.4 ± 12.7 ml/min/1.73m^2^ (95% CI: 80.0 to 84.8 ml/min/1.73m^2^) [29]. In a multiethnic study of 103 healthy volunteers from Singapore, participants with Indian ethnicity had lower measured GFR compared with other Asian populations (Chinese, Malay and others) [30]. This is comparable to mean mGFR of 79.44 ± 20.19 ml/min/1.73m^2^ seen in apparently healthy prospective renal donors in our population. The mGFR was <60 ml/min/1.73m^2^ in 9 participants. According to the 2012 KDIGO classification scheme, people with GFR <60 ml/min/1.73m^2^ would be deemed to have stage 3 CKD [1]. Low protein intake on account of largely vegetarian diet could be one of the reasons for comparatively low GFR in Indian population. Previous studies, however, have shown an acute rise in GFR with protein loading, indicating preserved renal functional reserve [29, 31]. An important question that needs to be answered is whether this low GFR in otherwise healthy individuals has long term adverse health consequences. Several meta-analyses have shown the adverse impact of low GFR on mortality and ESRD risk [32]. These meta-analyses, however, have not included any cohorts from India. The KDIGO classification [1] emphasizes that individuals should be labelled as having CKD only when the abnormality has *implications for health*. It remains unknown, however, whether having a low GFR in apparently healthy individuals in this population is physiological or carries an adverse prognosis as described in individuals with comparable eGFR levels elsewhere. Therefore, longitudinal studies that measure GFR and carefully and accurately define prognosis in this population are needed to inform the definition and staging of CKD. The Indian government has launched a scheme for a population wide non-communicable disease survey that includes assessment for CKD (http://timesofindia.indiatimes.com/india/Soon-door-to-door-screening-of-diseases/articleshow/53471865.cms). In the absence of an accurate equation, misclassification of healthy individuals as CKD could overwhelm the implementation of preventive and therapeutic strategies, and impair the credibility of the discipline [33]. This information is also important for selecting living kidney donors. Currently, only a minority of centers in India measure GFR, relying upon eGFR for donor selection. Reliance on equations that overestimate GFR could lead to inappropriate selection of donors with low true GFR, or inappropriate exclusion of healthy individuals who may not have any consequence from donation despite having a low GFR. This study is the first attempt at robust assessment of accuracy of currently recommended eGFR equations in Indian population. We have measured GFR by the gold standard method - urinary inulin clearance.

Our study has some limitations, including small sample size, limited regional representation (mainly North Indian population) and no formal measurement of muscle mass.

Our findings support the need for developing either a correction factor or a new equation for the Indian population. Such a study should be adequately powered, and should include participants from all geographies so that an equation that performs well across full spectrum of clinical characteristics in the population could be developed. Longitudinal studies are also needed for accurately estimating future risk of kidney failure, cardiovascular disease or mortality in populations who have had a baseline GFR measurement to validate the existing eGFR based diagnosis and classification system.

## Conclusion

In conclusion, our study shows that the existing creatinine-based GFR estimating equations significantly overestimate mGFR in the Indian population, which could be linked to the low muscle mass and vegetarian diet.

## Additional files

Additional file 1: Table S1. Estimating GFR equations. **Table S2.** Characteristics and measurements in study population (stratified by mGFR groups). **Table S3.** Performance of GFR estimating equations as compared to measured GFR by urinary inulin clearance (stratified by mGFR groups). **Table S4.** GFR measurements in study population (stratified by age). **Table S5.** Performance of GFR estimating equations as compared to measured GFR by urinary inulin clearance (stratified by age). **Table S6.** Performance of GFR estimating equations (eGFR calculated using serum creatinine values by modified Jaffe method) as compared to measured GFR by urinary inulin clearance. (DOCX 76 kb)

Additional file 2: Figure S1. A. Correlation between serum creatinine (mg/dL) measured by modified Jaffe’s method and enzymatic method (Pearson correlation coefficient, *r* = 0.965, *p* < 0.0001); B. Bland Altman analysis of difference between serum creatinine values (in mg/dL) measured by enzymatic and modified Jaffe’s methods (X-axis represents difference between two values and Y-axis represents mean of two values, red line represents mean difference and green line represents 95% limits of agreement. (DOCX 1387 kb)

## Abbreviations

CKD: Chronic kidney disease
CKD-EPI: Chronic kidney disease epidemiology collaboration
Cys: Cystatin C
eGFR: Estimated glomerular filtration rate
ESRD: End stage renal disease
GFR: Glomerular filtration rate
IDMS: Isotope dilution mass spectrometry
KDIGO: Kidney disease improving global outcomes
MDRD: Modification of diet in renal disease
mGFR: Measured glomerular filtration rate
RMSE: Root mean square error
SPSS: Statistical package for the social sciences
