# Supplementary material for: Existing creatinine-based equations overestimate glomerular filtration rate in Indians
Source: BMC Nephrol. 2018 Feb 1;19:22. doi: 10.1186/s12882-018-0813-9 (PMC5796440; doi:10.1186/s12882-018-0813-9)
Supplement: Supplementary file 1 — Estimating GFR equations. Table S2. Characteristics and measurements in study population (stratified by mGFR groups). Table S3. Performance of GFR estimating equations as compared to measured GFR by urinary inulin clearance (stratified by mGFR groups). Table S4. GFR measurements in study population (stratified by age). Table S5. Performance of GFR estimating equations as compared to measured GFR by urinary inulin clearance (stratified by age). Table S6. Performance of GFR estimating equations (eGFR calculated using serum creatinine values by modified Jaffe method) as compared to measured GFR by urinary inulin clearance. (DOCX 76 kb) [file 12882_2018_813_MOESM1_ESM.docx]

**SUPPLEMENTARY TABLES**

**Table S1: Estimating GFR equations**

| **eGFR equation**  **(ml/min/1.73m^2^)** | **Formula** |
| --- | --- |
| CKD-EPI_Cr_ | 141 x min (SCr/κ, 1)^α^ x max(SCr /κ, 1)^-1.209^ x 0.993Age x 1.018 (if female) |
| CKD-EPI (PK) | 0.686 x [141 x min (SCr/κ, 1)^α^ x max(SCr /κ, 1)^-1.209^ x 0.993Age x 1.018 (if female)]^1.059^ |
| CKD-EPI (JAP) | 0.813 x [141 x min (SCr/κ, 1)^α^ x max(SCr /κ, 1)^-1.209^ x 0.993Age x 1.018 (if female)] |
| MDRD | 175 x (SCr)^-1.154^ x (Age)^-0.203^ x (0.742 if female) |
| CKD-EPI_Cys_ | 133 x min (Scys/0.8, 1)^-0.499^ x max (Cys/0.8, 1)^-1.328^ x 0.996Age x  0.932 [if female] |

CKD-EPI: Chronic Kidney Disease Epidemiology Collaboration; Cys: cystatin C; GFR: glomerular filtration rate; JAP: Japan; MDRD: Modification of Diet in Renal Disease; PK: Pakistan; SCr: serum creatinine

κ = 0.7 (females) or 0.9 (males), α = -0.329 (females) or -0.411 (males)

**Table S2: Characteristics and measurements in study population (stratified by mGFR groups)**

| **Parameter** | **mGFR ≥60 ml/min/1.73m^2^ (n=54)** | **mGFR <60 ml/min/1.73m^2^ (n=76)** |
| --- | --- | --- |
| Age | 41.30±11.02 | 46.72±12.12 |
| Sex (M/F) | 21/33 | 51/25 |
| Height (cm) | 158.87±9.75 | 162.93±11.27 |
| Weight (kg) | 62.00±13.29 | 64.00±12.00 |
| BMI (kg/m^2^) | 24.54±4.75 | 24.68±9.20 |
| Hemoglobin (g/dL) | 12.60±1.00 | 13.70±2.33 |
| Serum creatinine (mg/dL) | 0.62±0.13 | 1.90±0.81 |
| Serum cystatin C (mg/dL) | 0.95±0.17 | 2.32±0.71 |
| Blood urea nitrogen (mg/dL) | 9.00±2.62 | 26.27±10.19 |
| Serum albumin (g/dL) | 4.34±0.36 | 4.20±0.43 |
| Measured GFR by urinary inulin clearance (ml/min/1.73m^2^) | 84.72±17.18 | 28.17±13.09 |
| Estimated GFR | | |
| CKD-EPI_Cr_ equation (ml/min/1.73m^2^) | 118.45±14.41^1^ | 46.83±24.35^3^ |
| CKD-EPI (PK) equation (ml/min/1.73m^2^) | 88.57±20.15^1^ | 31.46±16.72^3^ |
| CKD-EPI (JAP) equation (ml/min/1.73m^2^) | 107.75±13.87^1^ | 40.62±22.46^3^ |
| MDRD equation (ml/min/1.73m^2^) | 125.86±27.30^1^ | 43.63±22.46^3^ |
| CKD-EPI_Cys_ equation (ml/min/1.73m^2^) | 133.77±29.02^2^ | 46.37±24.13^4^ |

Expressed as mean ± standard deviation except sex. BMI: body mass index; CKD-EPI: Chronic Kidney Disease Epidemiology Collaboration; Cys: cystatin C; GFR: glomerular filtration rate; F: female; JAP: Japan; M: male; MDRD: Modification of Diet in Renal Disease; mGFR: measured GFR; PK: Pakistan

^1^significantly different from measured GFR by urinary inulin clearance (p<0.01 for each comparison)

^2^Not significantly different from measured GFR by urinary inulin clearance (p=0.281)

^3^significantly different from measured GFR by urinary inulin clearance (p<0.01 for each comparison)

^4^Not significantly different from measured GFR by urinary inulin clearance (p=0.187)

Mann-Whitney U test used for comparison between measured GFR and estimated GFR

**Table S3: Performance of GFR estimating equations as compared to measured GFR by urinary inulin clearance (stratified by mGFR groups)**

| **eGFR equation** | **Bias (mGFR-eGFR)**  **(ml/min/1.73m2)** | **95% Limits of agreement**  **(ml/min/1.73m^2^)** | **Precision (95% CI)**  **(ml/min/1.73m^2^)** | **Accuracy** | |
| --- | --- | --- | --- | --- | --- |
|  |  |  |  | **RMSE**  **(ml/min/1.73m^2^)** | **P_30_** |
| **mGFR ≥60 ml/min/1.73m^2^ (n=54)** |  |  |  |  |  |
| CKD-EPI_Cr_ | -33.73±15.84 | -64.77 to -2.68 | -38.03 to -29.41 | 37.20 | 22.2 |
| CKD-EPI_Cys_ | -3.85±19.19 | -41.46 to 33.76 | -9.09 to 1.39 | 19.41 | 81.5 |
| CKD-EPI (PK) | -23.02±15.65 | -53.69 to 7.65 | -27.29 to -18.75 | 27.76 | 50.0 |
| CKD-EPI (JAP) | -11.58±15.08 | -41.14 to 17.98 | -15.69 to -7.46 | 18.90 | 68.5 |
| MDRD | -41.14±24.66 | -89.46 to 6.54 | -47.87 to -34.41 | 47.84 | 22.36 |
| **mGFR <60 ml/min/1.73m^2^ (n=76)** |  |  |  |  |  |
| CKD-EPI_Cr_ | -18.67±15.28 | -48.61 to 11.27 | -22.16 to -15.17 | 24.06 | 22.4 |
| CKD-EPI_Cys_ | -3.30±10.54 | -23.96 to 17.35 | -5.71 to -0.89 | 10.98 | 69.7 |
| CKD-EPI (PK) | -12.45±13.69 | -39.28 to 14.38 | -15.58 to -9.32 | 18.44 | 31.6 |
| CKD-EPI (JAP) | -9.91±11.47 | -32.39 to 12.57 | -12.53 to -7.29 | 15.10 | 39.5 |
| MDRD | -15.63 to 13.75 | -42.41 to 11.49 | -18.61 to -12.32 | 20.63 | 28.94 |

CKD-EPI: Chronic Kidney Disease Epidemiology Collaboration; CI: confidence interval; Cys: cystatin C; eGFR: estimated glomerular filtration rate; F: female; JAP: Japan; M: male; MDRD: Modification of Diet in Renal Disease; mGFR: measured GFR; PK: Pakistan; P_30_: percentage of subjects with eGFR within ± 30% of mGFR; RMSE: root mean square error

**Table S4: GFR measurements in study population (stratified by age)**

| **eGFR equation** | **Age <50 years**  **(N=78)** | **Age≥50 years**  **(N=52)** |
| --- | --- | --- |
|  |  |  |
| mGFR | 43.80±23.56 | 56.89±35.37 |
| CKD-EPI_Cr_ | 82.75±45.41^1^ | 67.33±31.69^3^ |
| CKD-EPI_Cys_ | 62.59±38.27^2^ | 44.10±20.75^4^ |
| CKD-EPI (PK) | 74.41±42.53^1^ | 59.63±29.43^3^ |
| CKD-EPI (JAP) | 67.28±36.92^1^ | 54.74±25.76^3^ |
| MDRD | 85.53±53.63^1^ | 66.16±33.84^3^ |

CKD-EPI: Chronic Kidney Disease Epidemiology Collaboration; CI: confidence interval; Cys: cystatin C; eGFR: estimated glomerular filtration rate; JAP: Japan; MDRD: Modification of Diet in Renal Disease; mGFR: measured GFR; PK: Pakistan;

^1^significantly different from measured GFR by urinary inulin clearance (p≤0.01 for each comparison)

^2^Not significantly different from measured GFR by urinary inulin clearance (p=0.132)

^3^significantly different from measured GFR by urinary inulin clearance (p≤0.02 for each comparison)

^4^Not significantly different from measured GFR by urinary inulin clearance (p=0.723)

Mann-Whitney U test used for comparison between measured GFR and estimated GFR

**Table S5: Performance of GFR estimating equations as compared to measured GFR by urinary inulin clearance (stratified by age)**

| **eGFR equation** | **Bias (mGFR-eGFR)**  **(ml/min/1.73m2)** | **95% Limits of agreement**  **(ml/min/1.73m^2^)** | **Precision (95% CI)**  **(ml/min/1.73m^2^)** | **Accuracy** | |
| --- | --- | --- | --- | --- | --- |
|  |  |  |  | **RMSE**  **(ml/min/1.73m^2^)** | **P_30_** |
| **Age ≥50 years (n=52)** |  |  |  |  |  |
| CKD-EPI_Cr_ | -25.86±17.68 | -60.51 to 8.79 | -28.09 to -18.95 | 17.13 | 21.1 |
| CKD-EPI_Cys_ | -5.70±15.84 | -24.29 to 23.75 | -3.68 to 3.14 | 7.33 | 71.1 |
| CKD-EPI (PK) | -17.52±15.78 | -48.41 to 13.38 | -19.98 to -11.67 | 12.76 | 42.1 |
| CKD-EPI (JAP) | -10.38±13.17 | -36.19 to 15.43 | -14.56 to -7.30 | 9.88 | 51.3 |
| MDRD | -28.64±25.66 | -78.93 to 21.65 | -27.17 to -17.53 | 17.22 | 23.7 |
| **Age <50 years (n=78)** |  |  |  |  |  |
| CKD-EPI_Cr_ | -23.52±16.41 | -55.68 to 8.64 | -29.84 to -21.87 | 24.90 | 24.1 |
| CKD-EPI_Cys_ | -0.27±12.26 | -24.29 to 23.75 | -9.27 to -2.13 | 13.42 | 79.6 |
| CKD-EPI (PK) | -15.83±14.91 | -45.05 to 13.39 | -21.08 to -13.96 | 18.86 | 35.2 |
| CKD-EPI (JAP) | -10.93±13.04 | -36.48 to 14.63 | -13.35 to -7.41 | 13.58 | 51.9 |
| MDRD | -22.35±17.33 | 56.29 to 11.62 | -34.43 to -22.86 | 29.66 | 27.8 |

CKD-EPI: Chronic Kidney Disease Epidemiology Collaboration; CI: confidence interval; Cys: cystatin C; eGFR: estimated glomerular filtration rate; JAP: Japan; MDRD: Modification of Diet in Renal Disease; mGFR: measured GFR; PK: Pakistan; P_30_: percentage of subjects with eGFR within ± 30% of mGFR; RMSE: root mean square error

**Supplementary Table S6 - Performance of GFR estimating equations (eGFR calculated using serum creatinine values by modified Jaffe method) as compared to measured GFR by urinary inulin clearance**

| **eGFR equation** | **Bias (mGFR-eGFR)**  **(ml/min/1.73m^2^)** | **95% Limits of agreement**  **(ml/min/1.73m^2^)** | **Precision (95% CI)**  **(ml/min/1.73m^2^)** | **Accuracy** | |
| --- | --- | --- | --- | --- | --- |
|  |  |  |  | **RMSE**  **(ml/min/1.73m^2^)** | **P_30_ (%)** |
| CKD-EPI_Cr_ | -22.55±19.76 | -61.28 to 16.18 | -25.98 to -19.12 | 29.93 | 29.2 |
| CKD-EPI (PK) | -14.59±18.21 | -50.28 to 21.10 | -17.75 to -11.43 | 23.29 | 46.5 |
| CKD-EPI (JAP) | -8.67±16.32 | -40.66 to 23.32 | -11.50 to -5.88 | 18.43 | 53.5 |
| MDRD | -26.13±22.82 | -76.41 to 28.37 | -28.66 to -19.38 | 35.86 | 34.6 |

CKD-EPI: Chronic Kidney Disease Epidemiology Collaboration; CI: confidence interval; eGFR: estimated glomerular filtration rate; F: female; JAP: Japan; M: male; MDRD: Modification of Diet in Renal Disease; mGFR: measured GFR; PK: Pakistan; P_30_: percentage of subjects with eGFR within ± 30% of mGFR; RMSE: root mean square error
