# Supplementary material for: Existing creatinine-based equations overestimate glomerular filtration rate in Indians
Source: BMC Nephrol. 2018 Feb 1;19:22. doi: 10.1186/s12882-018-0813-9 (PMC5796440; doi:10.1186/s12882-018-0813-9)

**Figure S1: A.** Correlation between serum creatinine (mg/dL) measured by modified Jaffe’s method and enzymatic method (Pearson correlation coefficient, r= 0.965, p<0.0001)**; B.** Bland Altman analysis of difference between serum creatinine values (in mg/dL) measured by enzymatic and modified Jaffe’s methods (X-axis represents difference between two values and Y-axis represents mean of two values, red line represents mean difference and green line represents 95% limits of agreement)

**A.**

B.


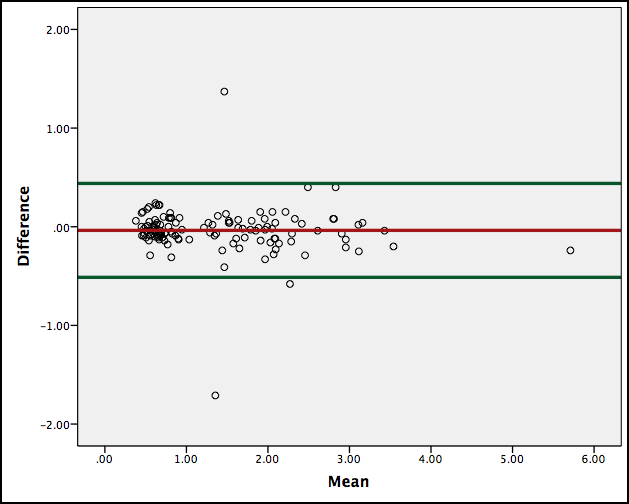

Supplement: Supplementary file 2 — A. Correlation between serum creatinine (mg/dL) measured by modified Jaffe’s method and enzymatic method (Pearson correlation coefficient, r = 0.965, p < 0.0001); B. Bland Altman analysis of difference between serum creatinine values (in mg/dL) measured by enzymatic and modified Jaffe’s methods (X-axis represents difference between two values and Y-axis represents mean of two values, red line represents mean difference and green line represents 95% limits of agreement. (DOCX 1387 kb) [file 12882_2018_813_MOESM2_ESM.docx]
